# Phenanthrene Degradation by Photosynthetic Bacterial Consortium Dominated by *Fischerella* sp.

**DOI:** 10.3390/life13051108

**Published:** 2023-04-28

**Authors:** José Martín Márquez-Villa, Juan Carlos Rodríguez-Sierra, Nayem Amtanus Chequer, Nubia Noemí Cob-Calan, José Quinatzín García-Maldonado, Santiago Cadena, Emanuel Hernández-Núñez

**Affiliations:** 1 Department of Industrial Biotechnology, CIATEJ-CONACyT, Zapopan 45019, Jalisco, Mexico; jmmv.ipn@gmail.com; 2 Department of Integral Institutional Formation, IPN-UPIIG, Silao de la Victoria 36275, Guanajuato, Mexico; 3 Department of Marine Resources, Centro de Investigación y de Estudios Avanzados del IPN, Merida 97310, Yucatan, Mexico; 4 Instituto Tecnológico Superior de Calkiní en el Estado de Campeche, Calkiní 24900, Campeche, Mexico

**Keywords:** cyanobacteria, PAH biodegradation, bacterial consortium, taxonomic classification, sequencing by synthesis

## Abstract

Microbial degradation of aromatic hydrocarbons is an emerging technology, and it is well recognized for its economic methods, efficiency, and safety; however, its exploration is still scarce and greater emphasis on cyanobacteria–bacterial mutualistic interactions is needed. We evaluated and characterized the phenanthrene biodegradation capacity of consortium dominated by *Fischerella* sp. under holoxenic conditions with aerobic heterotrophic bacteria and their molecular identification through 16S rRNA Illumina sequencing. Results indicated that our microbial consortium can degrade up to 92% of phenanthrene in five days. Bioinformatic analyses revealed that consortium was dominated by *Fischerella* sp., however different members of *Nostocaceae* and *Weeksellaceae*, as well as several other bacteria, such as *Chryseobacterium*, and *Porphyrobacter,* were found to be putatively involved in the biological degradation of phenanthrene. This work contributes to a better understanding of biodegradation of phenanthrene by cyanobacteria and identify the microbial diversity related.

## 1. Introduction

As the world population grows, crude oil demand and its derived products increase proportionally, so do oil spills and the inadequate disposal of industrial wastes, thus contributing to the ever-increasing environmental pollution [1,2,3]. Among the crude oil pollutant components, Polycyclic Aromatic Hydrocarbons (PAHs) are a ubiquitous class of environmental contaminants of great concern due to their toxicity, carcinogenicity, teratogenicity, and mutagenicity [4,5]. PAHs are highly recalcitrant molecules, and they are not easy to remove due to their hydrophobicity and low water solubility. Thus, it is possible to find them in crude oil, asphalt, creosote, tars, and waste products associated with wood preservatives [6,7]. The simplest PAH is naphthalene, because it has only two benzene rings and narrowly speaking is not polycyclic. Therefore, the increase in hydrophobicity and recalcitrance of these molecules is directly proportional to the increase in the number of rings [8] (Table 1).

Diverse PAHs have “bay/k” regions and it is well established that epoxides produced by these regions have high biological and chemical reactivity. PAHs’ environmental behavior and persistence vary according to their degree of solubility, the number of aromatic rings, molecular weight, concentration, and pollutant bioavailability, among others [10,11]. However, environmental factors, such as soil type, pH, temperature, pollutant time exposure, dissolved oxygen, salinity, nutrients, and water, can affect microbial activity which plays an important role in the persistence time of PAHs in the environment [12,13]. In addition, another factor that prolongs the persistence of PAHs in the environment is their association with heavy metals, where it is possible that the presence of heavy metals in the soil inhibits the cellular development of microbial communities [10,12].

Phenanthrene is a tricyclic aromatic hydrocarbon that can be found on sediments, terrestrial surfaces, and aquatic ecosystems [12,14,15,16]. It does not represent a hazard to human health because it has not shown genotoxic or carcinogenic effects, nevertheless, alterations in chromatin and inhibition of intracellular communication in Gap unions have been demonstrated [17]. The noncarcinogen phenanthrene is due to the simplest hydrocarbon that contains a bay region [18,19]. However, several studies have demonstrated phenanthrene toxicity in fish, algae, and superior organisms [18,19,20]. Phenanthrene is the simplest PAH that contains two regions, where epoxides are formed by the “k-region” which are highly carcinogenic [21,22]. It has been listed as 1 of the 16 PAHs of priority pollutants (Table 1) by USEPA [23]. So far, it is known that PAHs present in the environment could be removed by chemical and mechanical processes including evaporation by rotary tubular kiln, extraction by fluidized beds, removal of volatile fractions by rotary drum, chemical oxidation in reactors, direct pumping of the aquifer, elevation of hydrocarbons to the surface by flooding, adsorption by pumping on activated carbon and in situ leaching by surfactants [16]. However, for over two decades it has been demonstrated that the main and most useful process for PAH removal is through microbial processing and degradation [24]. It is well known that sediments with PAH contamination can be degraded by indigenous microorganisms that reside there [24,25]. 

Currently, in addition to the physicochemical methods of the decontaminating hydrocarbon-polluted matrices, hydrocarbon biodegradation is an emergent technology, and it is well recognized for its economical, efficiency, and security methods. Yet, the complex interaction between hydrocarbons, environment, and microbial community composition can make it relatively slow under normal conditions [2].

Cyanobacteria are the oldest phototrophic microorganisms and very versatile microorganisms, which, from natural ecosystems are able to develop microcosms under synergistic relationships with aerobic heterotrophic bacteria, where these interactions tend to be mutualistic [26,27]. They can thrive in diverse habitats, such as hot springs, Antarctic lakes and soils, and extreme euryhaline and eurythermal environments [28,29,30,31,32]. Due to this, previous studies indicate that holoxenic cultures have produced higher and more efficient biodegradation yields compared to axenic cultures [26,33,34], but despite the numerous studies mentioning the bacterial biodegradation of phenanthrene, virtually little is known about cyanobacteria and their enzymes involved in PAHs mineralization. These microorganisms can produce a wide variety of secondary metabolites. These compounds have been shown to be of industrial interest for their biological activity with pharmaceutical, nutraceutical, and agricultural potential as antitumor, anticancer, antiviral, antibiotic, and antifungal agents, among others [31,35,36]. Moreover, cyanobacteria have kindled great industrial interest, as their global product market is estimated grow from US 1547.23 million in 2020 to around US 2811.10 million in 2028; it is expected to grow at a CAGR of 7.9% from 2021 to 2028 [37].

Several ecological studies of microbial consortia have shown relevant improvements over the last 60 years, which were enhanced by the development of the polymerase chain reaction for the amplification of specific genes without the requirement of microbiological cultures. Thus, two decades ago, the proposed next-generation sequencing (NGS) opened the possibility to avoid traditional mutation detection methods to sequence multiple genes and unveil millions of variants simultaneously, as well as the minimal requirement of genomic material and consequently, the generation of several billions of nucleotides in a very short time and low cost [38]. Therefore, molecular characterization by massive sequencing of the 16S rRNA gene has shown great potential by introducing amplicons into next generation sequencing (NGS) to classify individual reads to specific taxa for the purpose of characterizing microbial consortiums [39,40,41,42].

As a matter of clarity, in this study we determined the biomass and biodegradation kinetics of phenanthrene using the cyanobacterium *Fischerella* sp. in consortium with aerobic heterotrophic bacteria and molecular characterization of the microbial community using the 16S rRNA gene sequencing by synthesis, which provided us with a deeper insight into the composition of the microbial consortium that is dominated by *Fischerella* sp. and that is capable of biodegrading phenanthrene as a model molecule.

## 2. Materials and Methods

### 2.1. Enrichment Culture, Incubation Conditions, and Photobioreaction System for Fischerella sp.

The cyanobacteria *Fischerella* sp. and its microbial consortia were collected and isolated from Cacahoatan, Chiapas region, Mexico. Moreover, the sampling point location (14°58′03.20″ N, 92°03′46.96″ W) was chosen with the presence of photosynthetic microorganisms developing over the sweetwater pond surface, on a site with no historical hydrocarbon pollution records. It is worth noting that samples were collected in triplicate using 250 mL sterilized Duran flasks (Schott, Mainz, Germany). The samples were transported in a cooler to the laboratory and stored at 25 °C at room temperature.

Taking a fresh sample from the isolation site, 1 mL of wet biomass was inoculated to the center of the 60 × 15 mm Petri dish (SYM laboratorios, Puebla, Mexico) in 0.7% *w*/*v* semi-solid BG-11 medium. Next, the cyanobacterial stock cultures on holoxenic conditions were inoculated with 1% *v*/*v* in 200 mL BG-11 liquid medium contained in 250 mL Duran flask (Schott, Germany) [43,44,45], with the final composition per liter being: C_6_H_11_FeNO_7_ 6 g, HNO_3_ 6 g, NaNO_3_ 1.5 g, K_2_HPO_4_∙3H_2_O 40 g, MgSO_4_∙7H_2_O 75 g, CaCl_2_∙2H_2_O 36 g, Na_2_CO_3_ 20 g, MgNa_2_EDTA∙H_2_O 1 g, and 1 mL of trace metal solution. The trace metal solution was composed of H_3_BO_3_ 2.86 g, MnCl_2_∙4H_2_O 1.81 g, ZnSO_4_∙7H_2_O 0.22 g, CuSO_4_∙5H_2_O 0.079 g, Na_2_MoO_4_∙2H_2_O 0.391 g, and Co(NO_3_)_2_∙6H_2_O 0.049 g. The pH value of the culture was kept at 7.1 ± 0.2 after autoclave (15 min at 121 °C under 100 kPa) and before use. The trace metal solution was added after sterilization of the macroelements to avoid precipitation of the components.

Cultures were incubated at 28 ± 1 °C with a photoperiod of 12:12 h (Light:Dark) employing cool-white fluorescent lamps (Electro Mag S.A. de C.V., Cuauhtémoc, Mexico) as artificial fluorescent illumination, with a continuous irradiance of 73 µmol/m^2^/s. Atmospheric air was supplied using an air compressor (Hagen, Mansfield, MA, USA) through a silicone hose (Cole-Parmer, Vernon Hills, IL, USA) with 1.5 vvm constant flux. The air was sterilized using Midisart 2000 (Sartorius, Göttingen, Germany) 0.20 μm air filters.

A scanning electron microscope (SEM) with an accelerating voltage of 25 kV (JEOL Ltd. Model JSM7600F, Tokyo, Japan) was used to visualize the morphology and cell structure of the cyanobacteria in greater detail. Samples were coated with gold to increase electrical conductivity.

### 2.2. Cell Growth Quantifications and Biomass Dry Weight Measurements

Cell growth quantifications were obatined through drying a membrane (Millipore, Darmstadt, Germany) of 0.22 μm, with a humidity balance PMB (Adam Equipment, Milton Keynes, UK) at 140 °C for 5 min. Next, excessive humidity on the membrane was removed by employing a desiccator (Bel-Art, Warminster, PA, USA) for up to 24 h and then weighted in an analytical balance (RADWAG Wagi Elektroniczne, Radom, Poland). The biomass was filtered via the KG 47 GLASS SUPPORT (Advantec MFS, Tokyo, Japan) with 300 mL and a Kitasato 1000 mL flask (KIMAX, Vineland, NJ, USA), using a vacuum pump (WACO, Nowon-gu, Seoul, Republic of Korea). The membrane with living biomass was dried out using the humidity balance PMB at 140 °C for 5 min and again excessive humidity was removed using a desiccator for 24 h, and weighed with an analytical balance. All samples were measured in triplicate.

The specific growth rate parameter (μ) associated with biomass development was determined considering the exponential phase through Equations (1) and (2) [46,47].

(1) $$\frac{dX}{dt}=\left(\mu \right)*\left(X\right)$$

(2) $$\int_{x_{0}}^{x_{t}}\frac{dX}{X}=\int_{t=0}^{t}\left(\mu \right)*(dt)$$

where, *dX*/*dt* represents the rate of biomass production in the culture at a given time and μ is the specific growth rate (1/day). Moreover, the duplication time (*t_d_*) can be expressed by Equation (3).

(3) $$t_{d}=\frac{Ln(2)}{\mu }$$

### 2.3. Phenanthrene Biodegradation Monitoring

In order, to determine the PAH mineralization rate, phenanthrene concentrations in the culture media were analyzed using a UV-Vis-NIR spectrophotometer (Agilent, Santa Clara, CA, USA). The concentration of phenanthrene was calculated from a standard curve based on peak area at 293 nm. The biodegradation process was determined using experiments carried out in triplicate, in a 250 mL Duran flask (Schott, Germany) with 200 mL of BG-11 liquid medium (pH 7.1 ± 0.2) employing the photobioreaction systems previously described in Section 2.1 of Materials and Methods. 

The experimental photobioreactors set up were treated with phenanthrene concentrations of 100, 50, and 10 mg/L, while the control experiments were left uninoculated and analyzed using the UV-Vis-NIR spectrophotometer described previously. For 5 days, the phenanthrene biodegradation rate was monitored every 24 h. All samples were measured in triplicate. The phenanthrene consumption was determined by Equation (4).

(4) $$-\frac{dS}{dt}=(q_{s})\times (X)$$

where, −*dS*/*dt* describes the rate of substrate consumption due to microbial activity at a given time, therefore, *q_s_* is the specific consumption rate of the substrate (1/day).

### 2.4. Genomic DNA Extraction and PCR Amplification of Variable Regions 3–4 of the 16S rRNA Gene

At the end of the experiment, samples were collected for further molecular analysis. DNA extraction was performed in duplicate using the DNeasy PowerSoil Kit (Qiagen, Venlo, The Netherlands) following the manufacturer’s protocol and a TissueLyser LT (Qiagen, The Netherlands) was used for cell disruption. DNA quality was evaluated by 1% agarose electrophoresis. The 16S rRNA gene amplicons were obtained using the universal bacterial primers SD-Bact-0341-bS-17 y SD-Bact-0785-aA-21 [48], spanning the V3 and V4 regions. PCR products were performed in duplicate and mixed in equal amounts for sequencing. Thermal cycling consisted of 3 min at 95 °C and 25 cycles of 30 s at 95 °C, 30 s at 55 °C, and 30 s at 72 °C, followed by 5 min at 72 °C. Amplicons were purified with the Agencourt AMPure XP beads (Beckman Coulter, Brea, CA, USA). PCR products were indexed using the Nextera XT Index, in accordance with Illumina’s protocol. PCR barcoded amplicons were again purified as described and then quantified using a Qubit 3.0 fluorometer (Life Technologies, Carlsbad, CA, USA). The correct size of the amplicons was verified using an Advanced QIAxcel (Qiagen, The Netherlands). Libraries were diluted with 10 mM Tris (pH 8.5) and pooled in equimolar concentrations (9 pM).

### 2.5. Illumina High-Throughput Sequencing and Bioinformatic Analysis

The final library was loaded onto the flow cell of the V3 MiSeq Reagent Kit (600 cycles), proceeding with a paired-end sequencing on the Illumina MiSeq platform (Illumina, San Diego, CA, USA) in CINVESTAV Merida. The demultiplexed resulting data were analyzed using the open-source bioinformatics pipeline QIIME2 (2017.11) [49]. The sequencing errors and their correction to properly resolve the amplicon sequence variants (ASV) were addressed via the DADA2 plugin, and chimeras were removed using the “consensus” method [50]. The taxonomic assignment of the representative’s ASVs was performed using the VSEARCH plugin [51], against the SILVA gene database (v. 132). A phylogeny was constructed from the resolved ASVs using the FastTree algorithm [52]. For a better understanding, in the next sections, we will refer to the determined ASVs as Operational Taxonomic Units (OTUs), but the ASV concept is a more precise alternative to the classical clustering based on a fixed percentage identity threshold [53]. Microbial community analysis and graphical outputs were performed in R (R Core-team, Vienna, Austria) using phyloseq [54], ggplot2 [55], and vegan [56] libraries.

### 2.6. Nucleotide Sequence Accession Number

The obtained 16S rRNA gene sequences were deposited in the NCBI archive under bioproject PRJNA562544.

### 2.7. Statistical Analysis

Statistical analysis was performed by means of a one-way analysis of variance (ANOVA). Tukey’s test was used in order to determine significant differences between all screening tests related to biomass growth and phenanthrene biodegradation (*p* < 0.0001), using the commercial software InfoStat.

## 3. Results and Discussion

### 3.1. Biomass Growth and Phenanthrene Biodegradation Monitoring

**Figure 1:**
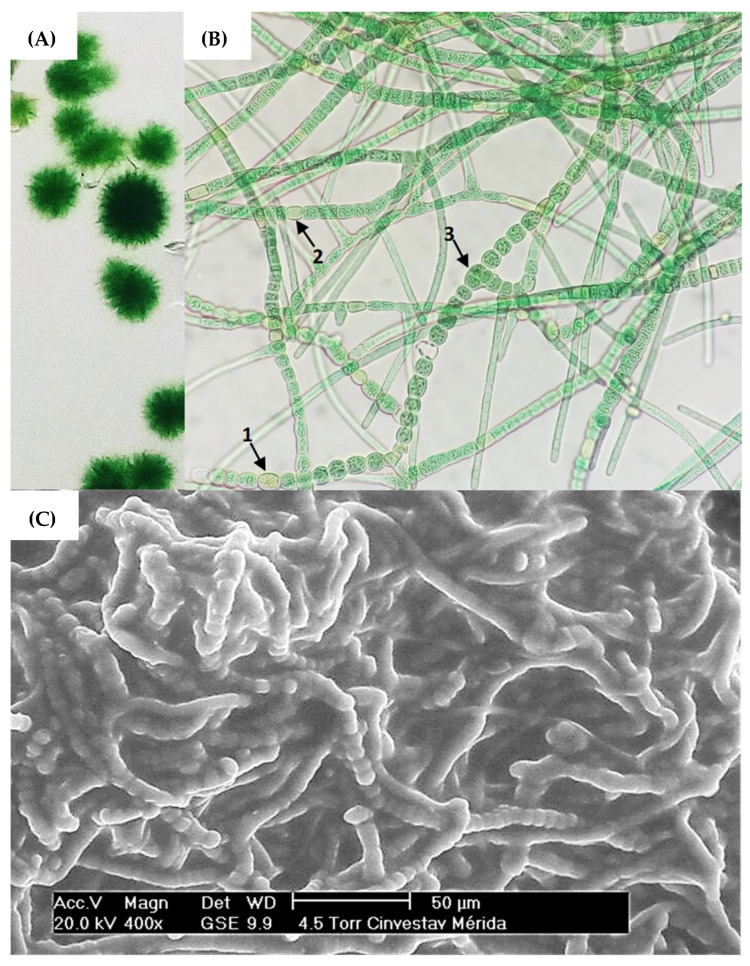
(**A**) Cyanobacteria colonies of strain *Fischerella* sp. on semi-solid agar cultures. (**B**) Brightfield microscopy with 40× objective, highlighting the morphological structures (1) acinetes, (2) heterocyst and (3) vegetative cell. (**C**) SEM microscopy of strain *Fischerella* sp.

The cyanobacteria strain was isolated from the sweetwater pond surface (Section 2.1) via enrichment in a semi-solid BG-11 medium (Figure 1A), therefore SEM microscopy showed that the strain had a filamentous structure. It was noticed that its characteristic filamentous structure is developed through communication channels between vegetative cells (hormogonium), heterocysts, and acinetes (Figure 1B). 

Vegetative cells can divide in more than one plane; i.e., they produce lateral branching to produce a mature trichome. Heterocysts can be found in terminal or lateral regions and hormogonia are composed of small cylindrical cells that enlarge and round into vegetative cells [32,35,57]. Based on this, ovoid vegetative cells approximately 7 μm in diameter were observed producing mature trichomes coiled amongst themselves (Figure 1B). The presence of mucilage, forming a secretion matrix coating the filaments, was also observed (Figure 1C), which has been described in research as a region that provides a phytosphere for bacteria in consortium with the cyanobacterial host [58]. Nonetheless, molecular identification via 16S RNA of the cyanobacterium *Fischerella* sp. and the microbial consortium was subsequently performed.

**Figure 2:**
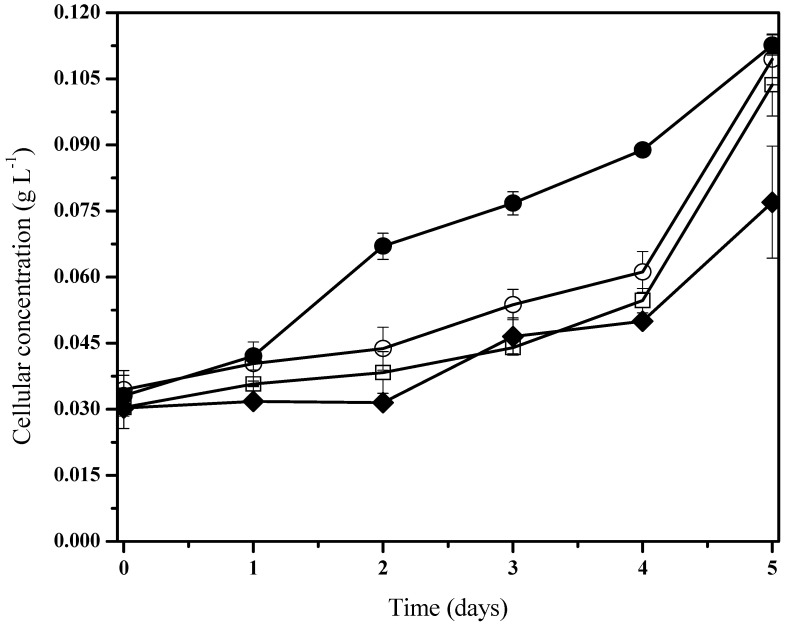
*Fischerella* sp. biomass behavior under holoxenic consortium during phenanthrene biodegradation. Pollutant concentrations are 100 mg/L (●), 50 mg/L (□), 10 mg/L (○), and control experiments (♦). Data shown as average and ± SD of triplicate determinations, significant differences between the groups (control group and phenanthrene concentrations were determined using one-way analysis of variance (ANOVA), significant difference existed between these experiments (*p* < 0.0001).

To demonstrate the biodegradation capacity of phenanthrene, the cyanobacteria consortium growth was measured for five days. Three different concentrations of pollutant (100, 50, and 10 mg/L) were employed in order to screen the experiment, and control tests were left uninoculated. It was observed that biomass growth was related to phenanthrene degradation as supplied on the medium as an additional source of carbon (Figure 2). By the fifth day, the microcosm behavior with phenanthrene showed a homologous tendency (Figure 2), which can be described as a low pollutant concentration detected by the chemotaxis mechanism from the bacterial consortium [59].

According to previous works [60,61] these results indicate that biomass growth kinetics from the presence of hydrocarbons can be fitted to the typical Monod’s kinetic, where adaptation phases are virtually nonexistent or reduced with relatively accelerated logarithmic phases. Moreover, it was noted in the cohort cells from control systems that there is an initial adaptation phase when compared to the group under phenanthrene treatment (Figure 2). The bacterial consortia presented a slight increase in their biomass development with pollutant exposition (Figure 2). Nevertheless, under the 50 mg/L phenanthrene concentration, there was a significantly higher specific growth rate when compared to the rest of the cultures with the presence and non-presence of phenanthrene (Table 2). It is to be noted that we characterized a mixed consortium of aerobic heterotrophic bacteria.

**Figure 3:**
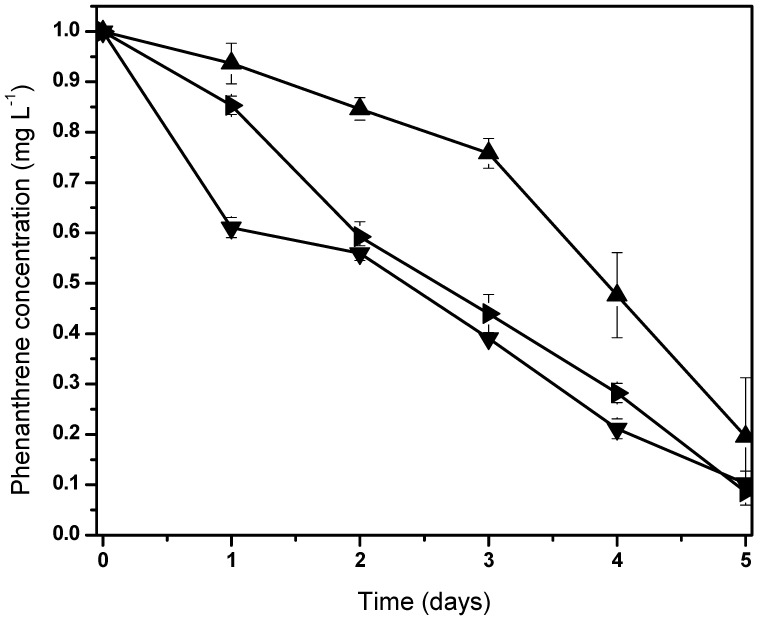
Normalized behaviors from phenanthrene biodegradation by *Fischerella* sp. under holoxenic consortium. Pollutant concentrations are 100 mg/L (▼), 50 mg/L (►) and 10 mg/L (▲). Data shown as average ± S.D. in triplicate, significant difference existed between these experiments (*p* < 0.0001). Control screen test data are not shown due to null variation in the phenanthrene concentration.

We hypothesized that these microorganisms possibly are related to *Fischerella* sp. in order to metabolize carbon compounds produced by the cyanobacteria [62]. The phenanthrene mineralization by the consortium was achieved almost totally after 5 days (Figure 3). Initial phenanthrene concentrations (100, 50, and 10 mg/L) were degraded to 89.7%, 91.3%, and 80.4%, respectively. All screening tests showed fluctuations with phenanthrene concentrations, where the highest PAH biodegradation was reached at 50 mg/L.

It is possible to associate several routes of PAHs consumption by microbial metabolism, as shown is various other studies related to bacterial, fungal, and algal biodegradation pathways [2,4,6,8,12,15]; however, the present research work focuses on the aerobic biodegradation of phenanthrene by bacteria. In general, reports have described that the basic mechanism of PAH biodegradation starts with the oxidation of the aromatic ring, followed by its scission, and ending in its transformation into biomass or molecules of linear structure, such as carbon dioxide and water [63]. Particularly, the biodegradation pathway of phenanthrene under aerobic conditions by bacteria starts with the enzymatic attack of phenanthrene dioxygenase at the 1,2- and 3,4-carbon atom positions to form cis-1,2-dihydroxy-1,2-dihydrophenanthrene and cis-3,4-dihydroxy-3,4-dihydrophenanthrene, where the main isomer is cis-3,4-dihydroxy-3,4-dihydrophenanthrene. Next, cis-3,4-dihydroxy-3,4-dihydrophenanthrene is oxidized to form 3,4-dihydroxyphenanthrene, which is subsequently cleaved to form 1-hydroxy-2-naphthoic acid, the main metabolite of the phenanthrene oxidative pathway which can be bifurcated depending on the enzymatic machinery present [64]. One catabolic pathway involves the hydroxylation of 1-hydroxy-2-naphthoic acid with the formation of 1,2-dihydroxynaphthalene, a compound that enters the naphthalene degradation pathway and subsequently metabolized to catechol, where the ortho- and meta-fission metabolites are transformed to succinate, acetyl-CoA, pyruvate, and acetaldehyde, which pass directly to the central pathways of cellular metabolism (Krebs cycle). On the other hand, the aromatic ring of 1-hydroxy-2-naphthoic acid is oxidized by the enzyme 1-hydroxy-2-naphthoate dioxygenase and forms another catabolic pathway, in which enzymatic reactions produce phthalic acid (phthalate) to form protocatechuic acid via the protocatechuate metabolic pathway and enter the Krebs cycle again [6,19,63,65]. 

Therefore, PAHs biodegradation can be performed from the whole-cell or biocatalytic approach. The use of the whole-cell approach involves the direct application of active microorganisms that use hydrocarbons as a carbon source, which means that the microorganisms secrete extracellular enzymes or degrade PAHs through their intracellular biocatalysts, however, this process is relatively slow due to predatory activity by other microorganisms and also due to the conditions of the contaminated site [66]. On the other hand, the biocatalytic approach has shown promising results in cases where low PAH concentrations are used and has the versatility of employing free or immobilized biocatalysts on surface cross-linked supports [67]. However, despite numerous studies on bacterial biodegradation of phenanthrene, currently little is known about cyanobacteria and the enzymes which catalyze the oxidation of PAHs. In addition, with the average kinetic parameters associated with phenanthrene biodegradation, it was possible to determine the specific substrate consumption rate and apparent biomass substrate yield (Table 2). It is evident through the fitting of the quantified data that the experiments with a PAH at 50 mg/L, achieved greater assimilation of phenanthrene. 

**Figure 4:**
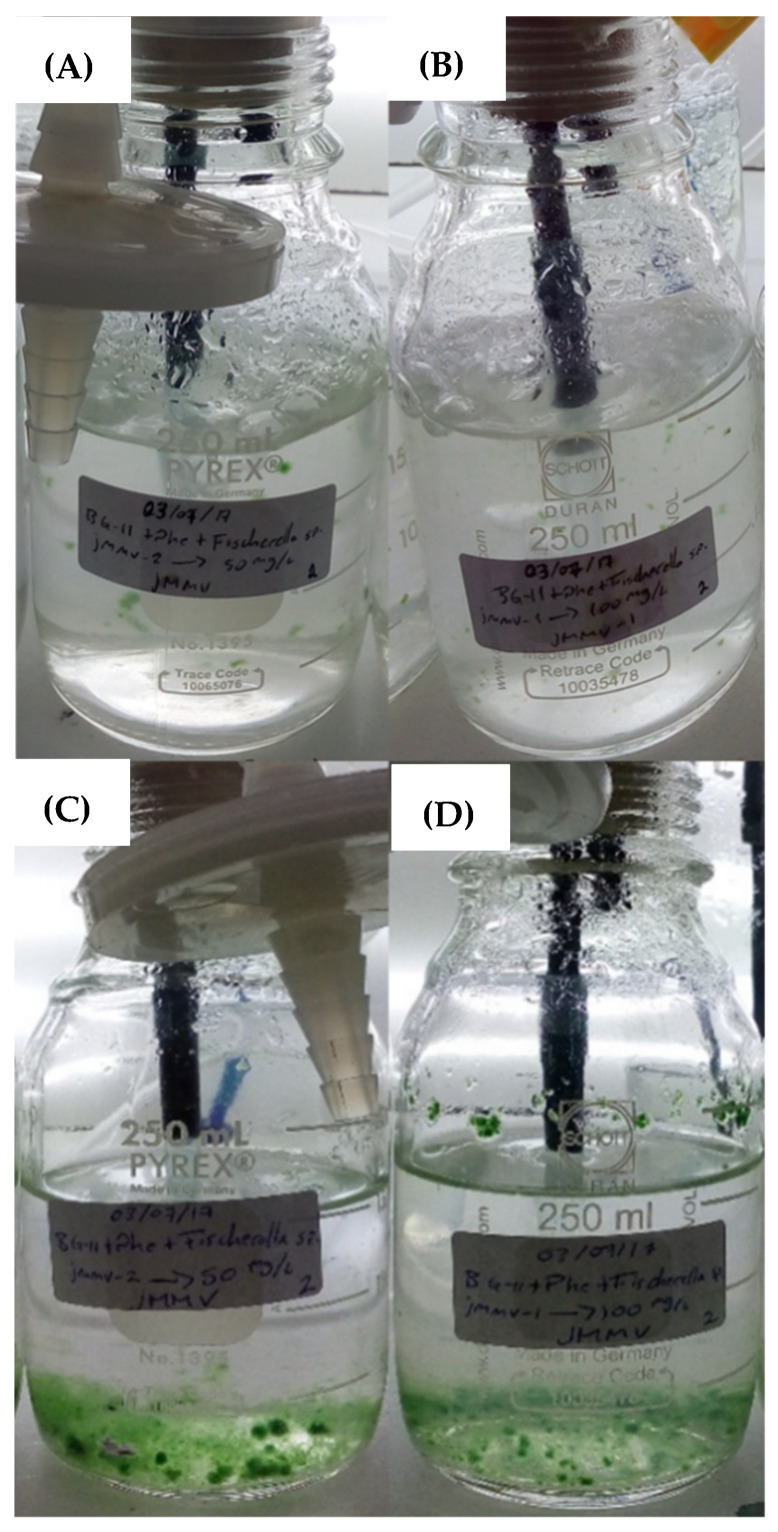
*Fischerella* sp. Cultures in photobioreactors. Cultures supplied with phenanthrene at 50 mg/L (**A**) and 100 mg/L (**B**) during the first day of growth and, cultures with phenanthrene at 50 mg/L (**C**) and 100 mg/ (**D**) after 4 days of operation.

On the other hand, during the preparation of the cultures, it was noticeable that the addition of phenanthrene generated turbidity in the liquid medium; however, after 4 days of culture, not only the cellular development of *Fischerella* sp. was visually noticeable, but also a clarification was visible in the culture medium. This provides a relatively clear evidence of PAH bioassimilation by the cyanobacterium *Fischerella* sp. And its microbial consortium (Figure 4). Cultures with higher turbidity due to phenanthrene concentration are displayed.

Interestingly, an alternative to exploring the degradation capabilities of the consortium presented in this work is the addition of amphiphilic molecules, either of the synthetic or biological source. It has been described that the use of surfactants in biodegradation studies of polycyclic aromatic hydrocarbons has generated wide interest [68,69]. Reports in the literature [70] have shown that even with the remarkable capacity of phenanthrene, and other PAHs in the synergism of the consortia, when applying biosurfactants into in vitro tests it was possible to reach up to 97% biodegradation in almost 30 days with the strain *Microbacterium esteraromaticum*; however, the rest of the isolated strains showed similar patterns in the enhancement of biodegradation. This alternative could be in contrast with the tests developed with the *Fischerella* sp. dominated consortium, taking into consideration that nearly 92% of phenanthrene biodegradation was achieved in five days.

Likewise, Table 2 shows average kinetic parameters associated with phenanthrene biodegradation, such as the specific rate of substrate consumption (*q_s_*) and the apparent biomass-substrate yield (*Y_xs_*) in the photobioreaction systems with phenanthrene concentrations. It is evident from the fit of the quantified data that cultures supplied with 50 mg/L achieved higher assimilation of phenanthrene to their metabolism. On the other hand, it is argued that cyanobacteria possess the capacity to oxidize hydrocarbons, so it is suggested to pursue further studies of *Fischerella* sp. to evaluate biodegradation activities under axenic conditions. However, based on several authors [71], it is mentioned that some microorganisms can metabolize polycyclic aromatic hydrocarbons as a carbon source, but due to the hydrophobicity of these compounds, they cross through the liquid interface where the active zone of the microorganisms is located at a slow rate.

### 3.2. Molecular Characterization of the Bacterial Consortium

**Figure 5:**
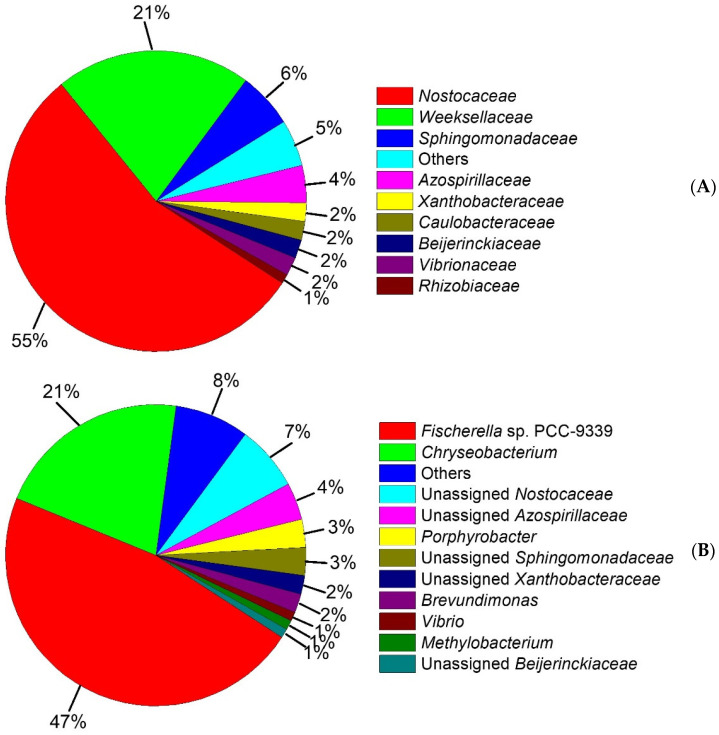
Taxonomic classification of the bacterial community. (**A**) Relative abundance of ≥1% from dominant families is shown, (**B**) Relative abundance of ≥1% from dominant genus is shown.

In this study to further examine the taxonomic composition of the microbial consortium, the Illumina sequencing of the 16S rRNA gene was performed and the results are shown in Figure 5. The bacterial community was distributed among 8 different families (Figure 5A) including *Nostocaceae* (55%), *Weeksellaceae* (21%), *Sphingomonadaceae* (6%), *Azospirillaceae* (4%), *Xanthobacteraceae* (2%), *Caulobacteraceae* (2%), *Beijerinckiaceae* (2%), *Vibrionaceae* (2%), *Rhizobiaceae* (1%) and others (5%). However, the most dominant families were *Nostocaceae* and *Weeksellaceae*. This result is in agreement with those reported in the literature [8,72,73,74,75], which suggested that *Weeksellaceae*, and other microbial families that we found, such as *Sphingomonadaceae*, *Xanthobacteraceae*, *Caulobacteraceae,* and *Rhizobiaceae,* are microbial families capable of degrading low molecular weight PAHs.

In this sense, the OTUs’ molecular characterization showed that the consortium was composed of 11 genera (Figure 5B) including *Fischerella* sp. PCC-9339 (47%), *Chryseobacterium* (21%), unassigned *Nostocaceae* (7%), unassigned *Azospirillaceae* (4%), *Porphyrobacter* (3%), unassigned *Sphingomonadaceae* (3%), unassigned *Xanthobacteraceae* (2%), *Brevundimonas* (2%), *Vibrio* (1%), *Methylobacterium* (1%), unassigned *Beijerinckiaceae* (1%) and others (7%). With this in mind, the consortium was composed of a higher proportion of microorganisms able to metabolize PAHs, whereas on previous reports [22,76,77,78] it has been described that *Chryseobacterium* belonging to the family *Weeksellaceae* is able to increase its cell development on polluted soils with crude oil, with the presence of carbazole as a sole source of carbon and energy, and on natural asphalts [79]. Moreover, the *Porphyrobacter* genus related to the family *Sphingomonadaceae* has been reported [80,81,82] to have mineralization capacity with naphthalene, 1-methylnaphthalene, acenaphthene, dibenzothiophene, phenanthrene, anthracene, biphenyl, dibenzofuran and bisphenol A on wastewater. *Xanthobacteraceae* is scarcely described with the capacity to metabolize naphthalene [83]. *Brevundimonas* and *Vibrio* genera belonging to *Caulobacteraceae* and *Vibrionaceae*, respectively, [84] have been previously reported with a high biodegradation capacity for crude oil and PAHs, such as phenanthrene, fluorene, pyrene, acenaphthene, anthracene, and fluoranthene, among others.

Nonetheless, *Methylobacterium* and *Beijerinckiaceae*, members of the family *Beijerinckiaceae*, have been explored previously [85,86] and shown to be capable of excessive growth in presence of monocyclic aromatic hydrocarbons and PAHs, with optimal behavior in the presence of naphthalene. 

To the best of our knowledge, *Fischerella* sp. PCC-9339 has no PAH biodegradation evidence and consequently, the results reported here are the first to describe its behavior under holoxenic conditions and with phenanthrene exposition as a model pollutant. Likewise, as we mentioned before in our study, *Nostocaceae* (55%) family was the most widely distributed. It is known that this cyanobacterium *Nostocaceae* with some genera, such as *Anabaena* sp. and *Nostoc* sp., can degrade aliphatic compounds contents of crude oil (decane, pentacosane, hexacosane, octacosane, and nonacosane) [87,88]. 

On the other hand, filamentous cyanobacteria, such as *Microcoleus chthonoplastes* and *Lyngbya aestuarii,* have been described in the literature [89] with the ability to develop biomass on crude oil contaminated sites; however, it is not yet defined whether cyanobacteria can play a direct role in the biodegradation of oil molecules. Our results suggest that the *Nostocaceae* family plays a main role during the process of biodegradation and bioavailability of phenanthrene.

## 4. Conclusions

This is the first study to report the phenanthrene biodegradation capacity of a heterotrophic aerobic bacterial consortium dominated by *Fischerella* sp. in order to determine the dynamics of the microbial process during PAH biodegradation. In light of the above discussion, phenanthrene mineralization behavior and biomass growth monitored during the five days of operation indicated phenanthrene consumption by microbial biodegradation. While the kinetic parameters were established as a function of biomass production and phenanthrene consumption as a substrate, these variables of the stoichiometric models described were based on the literature. Molecular characterization via Illumina sequencing of the 16S rRNA gene confirmed that the consortium includes several bacterial families reported in previous studies which are responsible for PAH biodegradation. However, as corroborated in this study and with other specific taxonomic studies, the *Nostocaceae* family has been shown to have a higher relative abundance with respect to the filamentous cyanobacterium *Fischerella* sp. and it offers clear indications as co-responsible for phenanthrene biodegradation. It is clear that the metabolic pathway of phenanthrene biodegradation described above could provide further insight into the biocatalytic nature of the cyanobacterium *Fischerella* sp. and of the bacteria present in the consortium. Therefore, a further quantification of the metabolic products would be key for a deeper understanding of this technology since it is virtually possible to produce, purify and immobilize its biocatalysts as a study target to deepen the oxidation of phenanthrene and other polycyclic aromatic hydrocarbons through an enzymatic format. In addition, it is important to isolate and characterize bacterial consortia from regions with a clear history of oil or hydrocarbon contamination to discover new microorganisms with more robust enzymatic mechanisms adapted to transform contaminants into simple molecules that are not harmful to natural matrices. Hence, this study provides results which provide a basis for future bioremediation studies with phenanthrene as a three-ring PAH model.

## Figures and Tables

**Table 1:** Properties of the 16 PAHs included in the USEPA list of priority pollutants [8,9,10].

Molecule	Rings Number	M. W. (g/mol)	Solubility in Water (mg/L)	Carcinogenic Factor
Naphthalene	2	128	31.7	0.001
Anthracene	3	178	0.07	0.01
Phenanthrene	3	178	1.3	0.001
Pyrene	4	202	0.14	0.001
Acenaphthene	3	154	3.9	0.001
Acenaphthylene	3	152	16.1	0.001
Fluorene	3	166	1.8	0.001
Fluoranthene	4	202	0.26	0.001
Chrysene	4	228	0.0006	0.01
Benzo[*a*]pyrene	5	252	0.0033	1
Benzo[*a*]anthracene	4	228	0.002	0.1
Benzo[*k*]fluoranthene	5	252	0.00055	0.1
Indeno[1, 2,3-*cd*]pyrene	6	276	0.062	0.1
Benzo[*b*]fluoranthene	5	252	0.0012	0.1
Dibenzo[*a*, *h*]anthracene	6	278	0.0005	5
Benzo[*g*, *h*, *i*]perylene	6	276	0.00026	0.01

**Table 2:** Cellular growth kinetic parameters from phenanthrene biodegradation.

Parameter	Phenanthrene Concentration			
	100 mg/L	50 mg/L	10 mg/L	0 mg/L
μ $\;(\frac{1}{day})$	0.22 ± 0.01	0.48 ± 0.03	0.44 ± 0.18	0.22 ± 0.03
$t_{d}\;(day)$	3.08 ± 0.20	1.44 ± 0.11	1.69 ± 0.68	3.16 ± 0.49
*Y_xs_* ($\frac{g\;Fischerella\;sp.}{mg\;Phenanthrene}$)	0.0013 ± 0.0006	0.0010 ± 0.00007	0.0162 ± 0.0103	-
*q_s_* ($\frac{1}{day}$)	186.66 ± 13.70	1133.70 ± 48.06	59.43 ± 38.82	-
